# Advance in Pharmaceutical and Chemical Science

**Published:** 1877-07-20

**Authors:** William A. Green

**Affiliations:** Macon, Ga.


### ADVANCE IN PHARMACEUTICAL AND CHEMICAL SCIENCE.

By WILLIAM A. GREEN, M. D., Macon, Ga.

No science has been cultivated with more difficulty than that of medicine. Its progress has been gradual, and, therefore, more likely to be eventually permanent. While political speculations are daily becoming more uncertain, in their operations, thetriumphs of intellectual superiority over prejudice, in our profession, is everywhere apparent. In our own country, more than any other, unjust disabilities are being abolished, and the gates of learning thrown open to every candidate. Every reader of medical history knows how fearfully we have had to contend, in turn, with the powers of witchcraft and priestcraft that sought to monopolize our practice as a privilege from the gods, and with the furious opposition of cotemporary members of the profession, whose capacity and vanity were alarmed by the introduction of novel doctrines and remedies which they were too old, too busy, or too obstinate to learn or investigate. Aristippus very properly replied to a man who boasted of his reading: "It is not those who eat the most that are hale and hearty, but those who can best digest." Hence the distinction between the philosophical physician and the mere dogmatizer. The one is guided by the observation of facts, the other by glossorial success. Men of erudition are seldom men of genius. The exploring mind is ever anxious to take flight from the prison-house of scholastic restraints.

In these papers on modern pharmaceutical science I propose writing, I merely rank myself a *compiler*. Subjects of great importance I shall only sketch, sometimes, it may be, with too fanciful a pencil, which, by being thus popularized, may induce abler hands to embody in a more permanent, attractive, and instructive form. The variety of matter will oblige me to be discursive, and to have recourse to some subjects not easily abridged. If I hold up error and evil to exposure I will not, I hope, be influenced by hostility toward men or firms, ranks or creeds. If I unwillingly or unwittingly give offence, I shall most sincerely lament it. My material will be gleaned from the works and writings of cotemporaries, well known, justly appreciated, and in all respects fully relied upon, as well as my own experience in a practice of a quarter of a century, four years of which was spent in the tented field, following the grand old army of Northern Virginia, in every march and *every battle*, under her illustrious commander, R. E. Lee.

Without further preface I will proceed to discuss the properties and merits of comparatively a new remedy or compound of great therapeutic value—

"SEVEN SPRINGS IRON AND ALUM MASS,"

a compound ferruginous preparation procured from the mineral waters of the celebrated "Seven Springs" situated in Washington county, Virginia, and manufactured by Messrs. Landrum & Litchfield, of Abingdon, Va. It is obtained by conducting the water from the springs into boilers on a furnace, where by means of a strong heat it is evaporated, leaving the combined mineral substance in a condensed mass, which is called the "*Seven Springs Iron and Alum Mass.*" Its medicinal properties are tonic, alterative, diuretic, and anti-periodic. It is no more a *nostrum*, patent, or proprietary medicine than castor oil, bi-carb. soda, or Congress water, and consequently there should be no *prejudice* in the minds of physicians in prescribing it.

Prof. J. W. Mallett, of the University of Virginia, has made an analysis of a number of bottles, which is published in a pamphlet that can be found in the principal drug stores, or procured from the proprietors at Abingdon, Va. I have for many years been constantly prescribing it, perhaps as often as any other medicine of like properties in the materia medica, and with as uniform success. It is emphatically one of nature's own remedies, and, I am confident, honestly and skilfully prepared. I use it very satisfactorily in most cases of disordered liver, quite common in this malarial climate, where the blood is impoverished and broken down in its invigorating properties by repeated attacks of chill and fever; in dyspepsia, rheumatism and neuralgia, anæmia and chlorosis. I have had special good results from it in chronic skin diseases, in indolent ulcers, applied both locally on the ulcerated surface as a dressing, and constitutionally. In the headaches which I have often thought our people in this section of the country suffer with more than any other, it is quite a familiar remedy. The following extract of a letter from Mr. John Ingalls, one of our leading druggists and an educated pharmaceutist and chemist, will give an idea of how the medicine is esteemed in this city: "I have been selling the *Ferruginous Mass* since 1871, constantly. The sale has been excellent, considering no effort was or has been made to bring it prominently before the people by advertising or otherwise. I have *invariably had most excellent reports from it*, and consider it a most *valuable medicine.*" Its harmlessness and convenience of administration, as well as being very palatable, will always make it acceptable as a household remedy. The dose is from ten to fifteen grains as an astringent, from forty to sixty grains as a tonic, alterative, and diuretic, given in pill form to adults, and in syrup to children. The following cases from my note-book will better illustrate its efficacy and curative properties than anything I could assert concerning it, and I have many such. But these two are sufficient and pointed cases.

### HÆMATEMESIS, VICARIOUS MENSTRUATION, AND AGGRAVATED HYSTERIA.

Mary H., aet. 19, came under my care May, 1875. Enjoyed good health until sixteen years of age, when, she said, she had a convulsion followed by brain fever, and on recovery vomited blood for three days successively, at regular monthly periods, and if this did not occur she had pains between her shoulders, at the epigastrium, and dyspnœa. This vomiting of blood continued regularly for three (3) years, but she never menstruated properly. Past nine months the discharge has ceased, and three months before consulting me had a severe hysterical or epileptic fit. Appears stout and well nourished, but prostrate; tongue dry and brown; lays motionless in bed, and refuses food. Complains of pain in lower part of back and groins; abdomen tympanitic and distended; no disease of uterus; pulse feeble and very quick. Milk was poured into her mouth and she was compelled to swallow it, and sometimes brandy and cream, thus receiving considerable nourishment. She had been variously treated, being able to employ the best medical attention the State afforded. Had taken galbanum, zinc, aloes and myrrh, mercury, colocynth, and henbane, the bromides, quinine and steel, with wine and cod liver oil, together with counter irritation and local depletion. I was certainly satisfied she had already had the full benefit of the usual and unusual treatment in such cases, and scarcely knowing what more to recommend, I prescribed the "Iron and Alum Mass, an astringent, tonic, and alterative being clearly indicated in her case. I directed forty grains three times a day, at meal times, with bromide potass. grs. xxx. morning and night, in glass of water, together with full and liberal nourishment. In one week saw her again, stomach retains food, and can get up in bed. At end of four weeks could ride in her carriage an hour or two, and in two weeks more seemed perfectly convalescent, with instructions to continue the mass in gradually reduced doses until permanently restored to health. At this writing she is well, and advises every sick woman in her vicinity to take "iron and alum mass."

CASE II.—DYSPEPSIA—PYROSIS.

A gentleman (Oct., '53) applied to me, for medical advice; his mind had been much overworked, and for more than ten years had suffered exceedingly; as much, or more, from mental depression, than actual disease. He was a most enthusiastic Methodist preacher, with a large and dependent family. Whilst his energies were being overtasked, he experienced sudden vomiting, and nearly every day water was regurgitated into his mouth. After two months he applied to a physician, but his symptoms increased in severity, and were associated with languor and exhaustion. He gave up his ministerial work and traveled. Yet his ailment continued; the sudden severe pain at his stomach was only relieved by lying on his back; he could obtain but little rest, and suffered occasional vertigo. He returned home and gave up in despair. The fluid ejected was tasteless, clear, and like water; it was generally brought up three hours after eating; his nights were wretched. Various forms of medication and diet had been tried—prussic acid, bismuth, silver, nitric and other acids; gentian, soda, columbo, etc., without apparent benefit. His countenance was natural, but his mind dejected; the conjunctiva watery, the tongue clean, the circulation feeble, the pulse compressible, the urine acid. Sp. gr. 1020, not albuminous, neither did it contain crystals, nor deposit; nothing could be detected on palpation of the abdomen; but slight pain was produced at the scrobiculus cordis.

I prescribed steel and quinine, with capsicum and conium, and a sedative draught at night, for two or three weeks, with but little benefit, when, finally, being tired and worried with his *constant* and *punctual* attendance at my office hours, and despairing of doing him any good, anxious to get him off my hands, though exceedingly sorry for him, I concluded to make a trial of the "*Iron and Alum Mass,*" encouraging him with the information that I had *at last* discovered a *panacea* for his troubles, and if he would take it *religiously*, and stick to a prescribed diet, which I would put in writing, and with a change of climate, I was confident he would recover. I at once saw a gleam of hope in his cast-down face. He promised faithfully, when I ordered "the Mass" in forty grain doses, three a day, at meal times, and a sedative draught at bed time, and sent him on his way. I was gratified, three months after, to find him almost perfectly well. He re-joined the Conference, (having been put on the superannuated list,) and to-day is proclaiming the gospel to a large flock, and losing no opportunity of prescribing the "Iron and Alum Mass," for all who are "*sick in body.*"

I have selected these two cases because the indications in each were clear and unmistakable, as requiring the properties claimed for "the Mass." I now hope my professional brethren will give it a trial, and I will vouch for a good result. I am really surprised that it has not been more generally used by them. I believe the non-professional are better acquainted with it than the physicians, from what our druggists tell me.

